# Dexmedetomidine Reduces Shivering during Mild Hypothermia in Waking Subjects

**DOI:** 10.1371/journal.pone.0129709

**Published:** 2015-08-03

**Authors:** Clifton W. Callaway, Jonathan Elmer, Francis X. Guyette, Bradley J. Molyneaux, Kacey B. Anderson, Philip E. Empey, Stacy J. Gerstel, Kate Holquist, Melissa J. Repine, Jon C. Rittenberger

**Affiliations:** 1 Applied Physiology Laboratory, Department of Emergency Medicine, University of Pittsburgh, Pittsburgh, PA, United States of America; 2 Department of Critical Care Medicine, University of Pittsburgh, Pittsburgh, PA, United States of America; 3 School of Pharmacy, University of Pittsburgh, Pittsburgh, PA, United States of America; Azienda Ospedaliero-Universitaria Careggi, ITALY

## Abstract

**Background and Purpose:**

Reducing body temperature can prolong tolerance to ischemic injury such as stroke or myocardial infarction, but is difficult and uncomfortable in awake patients because of shivering. We tested the efficacy and safety of the alpha-2-adrenergic agonist dexmedetomidine for suppressing shivering induced by a rapid infusion of cold intravenous fluids.

**Methods:**

Ten subjects received a rapid intravenous infusion of two liters of cold (4°C) isotonic saline on two separate test days, and we measured their core body temperature, shivering, hemodynamics and sedation for two hours. On one test day, fluid infusion was preceded by placebo infusion. On the other test day, fluid infusion was preceded by 1.0 μg/kg bolus of dexmedetomidine over 10 minutes.

**Results:**

All ten subjects experienced shivering on placebo days, with shivering beginning at a mean (SD) temperature of 36.6 (0.3)°C. The mean lowest temperature after placebo was 36.0 (0.3)°C (range 35.7-36.5°C). Only 3/10 subjects shivered on dexmedetomidine days, and the mean lowest temperature was 35.7 (0.4)°C (range 35.0-36.3°C). Temperature remained below 36°C for the full two hours in 6/10 subjects. After dexmedetomidine, subjects had moderate sedation and a mean 26 (13) mmHg reduction in blood pressure that resolved within 90 minutes. Heart rate declined a mean 23 (11) bpm after both placebo and dexmedetomidine. Dexmedetomidine produced no respiratory depression.

**Conclusion:**

Dexmedetomidine decreases shivering in normal volunteers. This effect is associated with decreased systolic blood pressure and sedation, but no respiratory depression.

## Introduction

Induced mild hypothermia is a potential therapy for many diseases. Reducing core body temperature may improve brain recovery after cardiac arrest and traumatic brain injury [1,2] Preclinical data and small trials suggest that mild hypothermia reduces ischemic injury during myocardial infarction [3–6]. Induced hypothermia is feasible after acute stroke, and larger trials of hypothermia after stroke and intracranial hemorrhage are underway [7–12]. While manipulating temperature is relatively easy in patients with coma, waking patients shiver when core body temperature declines below about 36.5°C impeding or preventing sustained hypothermia [13, 14] Sustained hypothermia in waking patients is thus unpleasant and requires greater heat transfer unless shivering is suppressed. Development and refinement of a shivering suppression protocol is required as shivering prevents induction and impairs the maintenance of hypothermia.

Various drugs decrease the threshold temperature for shivering, and deep sedation or general anesthesia can completely eliminate shivering. However, deep sedation is not desirable for many populations including patients with acute stroke. Prior studies suggest that the alpha-2 adrenoceptor agonist dexmedetomidine reduces shivering without excessive sedation [15, 16] Dexmedetomidine is useful for sedation of select patients in the intensive care unit and for procedural sedation in pediatrics or special populations where respiratory compromise is undesirable [18–20] Dexmedetomidine has also been used to control the hyperadregergic response in stimulant overdoses, to treat sympathetic storming, and to prevent development of malignant hyperthermia [21–23] These observations suggest that dexmedetomidine might provide a simple pharmacological intervention to suppress shivering and improve comfort without producing respiratory compromise in patients with ongoing stroke or other acute ischemia.

In order to maximize the potential benefit of hypothermia during ongoing ischemia, temperature should be decreased as quickly as possible, perhaps even prior to hospital arrival. Interventions to suppress shivering should be simple, fast, and long lasting. Available studies used computer-driven infusions of dexmedetomidine and do not describe the detailed time course of effects or efficacy of bolus administration of dexmedetomidine, which would be easier for administration outside of the hospital or ICU. Therefore, we tested whether a single bolus of dexmedetomidine would reduce the shivering threshold in normal volunteers [15–17]. We also measured the duration of effects of the drug on comfort, sedation and hemodynamics. Furthermore, we measured the plasma levels of drug to allow comparison with other studies. This study was designed to test the specific hypothesis that bolus administration of 1.0 μg/kg of dexmedetomidine would reduce the shivering threshold by at least 1.0°C.

## Methods

### Ethics Statement

The Institutional Review Board of the University of Pittsburgh approved this protocol. All subjects provided written informed consent prior to participation. The experimental design was a crossover in which each subject was tested on two days separated by at least one week. On one test day, we administered placebo and on the other day we administered dexmedetomidine (Hospira, Lake Forest, IL). The study drug was stored in the standard clinical manner and was provided by the Investigational Drug Service for each protocol day. The order of drug and placebo was assigned for each subject according to a random number generated by a computer, and subjects were blinded to the order. During our initial application for IRB approval, a reviewer expressed a safety concern that the investigators were blinded, thus investigators were not blinded. For each protocol a standard airway kit equipped with bag valve mask, endotracheal, and supraglottic airways was in the laboratory. Standard advanced cardiac life support medications and an automatic external defibrillator was also in the laboratory.

Subjects were adults (age >18 years) recruited from the University community, self-reported to be free of heart disease, vascular disease, renal impairment, hepatic impairment, and allergy to dexmedetomidine. Subjects were excluded for history of impaired gastrointestinal motility or abdominal surgery because these are contraindications to the core temperature capsule. Women could not be pregnant or breastfeeding. On a screening visit, subjects provided a brief medical history, and we obtained an electrocardiogram to exclude the presence of conduction abnormalities. These include a QRS>120msec, evidence of Brugada or Wolf-Parkinson White abnormalities. Resting vital signs were confirmed to be normal (baseline temperature >36.5°C and heart rate ≥50 beats per minute (bpm), systolic blood pressure (SBP)≥100 mmHg and ≤160 mmHg.). Women were confirmed to have negative urine pregnancy tests on each test day.

On test days, subjects refrained from eating or drinking for 6 hours prior to testing. At least one hour prior to testing, subjects swallowed an ingestible capsule that measures internal temperature (CorTemp, HQInc., Palmetto, FL). This capsule transmits gastrointestinal temperature data to a receiving unit continuously as a measure of core temperature [24]. An 18g or 20g intravenous catheter was inserted into the antecubital vein bilaterally. Monitors were placed to measure heart rate, respiratory rate, and pulse oximetry. End-tidal carbon dioxide was measured via nasal cannula. Automatic non-invasive blood pressure was recorded every 4 minutes during the protocol. Experiments were conducted between 8 am and 2 pm with subjects lightly clothed in a laboratory with ambient temperature 20–22°C.

Subjects rated thermal sensation and comfort every five minutes using qualitative scales. Comfort was assessed with a general (overall) comfort scale anchored by 1, “comfortable,” and 4 “very uncomfortable”. Thermal burden was anchored by 1, “comfortable,” and 5 “very hot.” Every five minutes, investigators rated subject sedation using Richmond Agitation and Sedation Scale (RASS) and shivering using the Bedside Shivering Assessment Scale (BSAS) [25–27]. The RASS is a 10-point scale ranging from -5 (unresponsive to voice or painful stimulation) to 4 (combative and danger to self or staff). It is commonly used in assessing sedation in the intensive care unit setting. The BSAS is a 4-point scale used to determine the severity of shivering. It ranges from 0 (no shivering) to 3 (severe shivering- generalized or sustained upper/lower extremity shivering).

Testing (time = 0) began with an infusion of 1.0 μg/kg dexmedetomidine diluted in 70 ml of normal saline (NS) or a placebo infusion of 70 ml of NS over 10 minutes (Baxter Healthcare, Deerfield, IL; pH 5.0). Hypothermia induction with cold NS started 5 minutes after the start of the dexmedetomidine infusion. For cooling, we rapidly infused 30 ml/kg, rounded to the nearest liter, not to exceed 2 L, of cold (4°C) 0.9% NS using a pressure bag (about 10 min/L). Investigators stopped drug administration if heart rate declined to less than 45 bpm for 2 consecutive minutes, SBP declined to <90 mmHg for 5 consecutive minutes, tissue pulse oximetry (SpO2) declined to <90% for one minute, or the subject became deeply sedated (RASS -4).

After the NS infusion, we monitored subjects for a minimum of 120 minutes, and until RASS was 0 and temperature was ≥36°C. At 10, 30, and 60 minutes after the drug infusion, we obtained 5 ml of blood from the intravenous catheter for measurement of plasma dexmedetomidine levels.

Blood was collected in citrated tubes and centrifuged at 1000xg for 10 minutes. Plasma was decanted into separate tubes stored at -70°C until processing. We measured plasma dexmedetomidine concentrations using an ultra-performance liquid chromatography tandem mass spectrometry (UPLC-MS/MS) method modified from Li et al [28] Dexmedetomidine was extracted from 0.2 mL of plasma using 5 mL of diethyl ether containing ondansetron (50ng/mL) as an internal standard, evaporated under nitrogen gas, and reconstituted in 0.2 mL 70:30 0.1% formic acid:acetonitrile. Separation (7.5μL) was achieved using a C18 1.7 m, 2.1x150 mm column and a mobile phase consisting of 70:30 0.1% formic acid:acetonitrile run at 0.3 mL/min (Acquity, Waters, Milford, MA). Mass spectrometric detection of dexmedetomidine and ondansetron was performed simultaneously using a TSQ Quantum ultra triple quadrupole mass spectrometer (Thermo, Waltham, MA) operated in positive electrospray ionization mode. Eight calibration standards (10, 20, 50, 100, 500, 1000, 2000, and 5,000 pg/mL) and 3 quality control samples 25, 200, and 2500 pg/mL were prepared in 0.2 μL blank plasma. Calibration curves were linear from 10–5,000 pg/mL (R^2^>0.9961) and the lower limit of sample quantification was 10 pg/mL. Inter- and intra-assay variation was less than 8.9%. Plasma concentrations were quantified from the standard curve using dexmedetomidine/internal standard area ratio and diluted as necessary to be within the validated assay range.

### Statistical Analysis

Shivering threshold was determined as the core temperature when shivering was visible (BSAS>0). After confirming normal distributions of the data, the shivering threshold for each subject was compared between placebo and dexmedetomidine days using paired t-tests. Physiological data other than SpO2 were normally distributed and compared using general linear models. In order to account for the repeated measures and clustering of data within subjects, we performed time-course analyses using the longitudinal data modules of STATA 13.0 (College Station, TX). Exploratory analyses found no significant interactions between subject and time, subject and drug, or subject, drug and time, and the pattern of results was identical in all analyses. Therefore, we did not include these interaction terms in final models, but we did include the interaction of drug and time. The distribution of SpO2 was not normal, and we compared the minimum observed SpO2 between drug and placebo for each subject as matched pairs using Wilcoxon rank-sum test. Ordinal scales (BSAS, RASS, Comfort and Thermal) were summarized as the maximum, minimum, and the sum of all scores (area-under-the-curve) for each testing session. We compared ordinal parameters between drug and placebo days as matched pairs using Wilcoxon sign-rank test. We selected an alpha-error rate of 0.05 for analyses, which we conducted using STATA 13.0 (College Park, TX).

In order to select our sample size, we defined a change in shivering thresholds of at least 1°C to be clinically interesting. Previous studies of shivering thresholds in normal volunteers found a standard deviation of 0.5°C [29]. Assuming a within subject correlation of 0.3, a standard deviation of 0.5°C, and α = 0.05, seven subjects would provide 84% power to detect a change of 0.5°C. To minimize uncertainty, we selected ten subjects, which would provide 99% power to detect a change of 1.0°C.

## Results

Eleven subjects were screened for this study, and one was excluded based on his medical history. Ten subjects (8 men) started and completed the protocol. These subjects mean weight was 75.3 (SD 10.9) kg and mean age was 28 (SD 6) years. Nine subjects received all of the drug infusion. In one subject, the drug infusion caused dizziness and nausea during which his heart rate declined to <40 bpm. For this subject, dexmedetomidine infusion was stopped after infusion of 65 ml of 70 ml (0.93 μg/kg). Symptoms resolved within 5 minutes.

**Fig 1:**
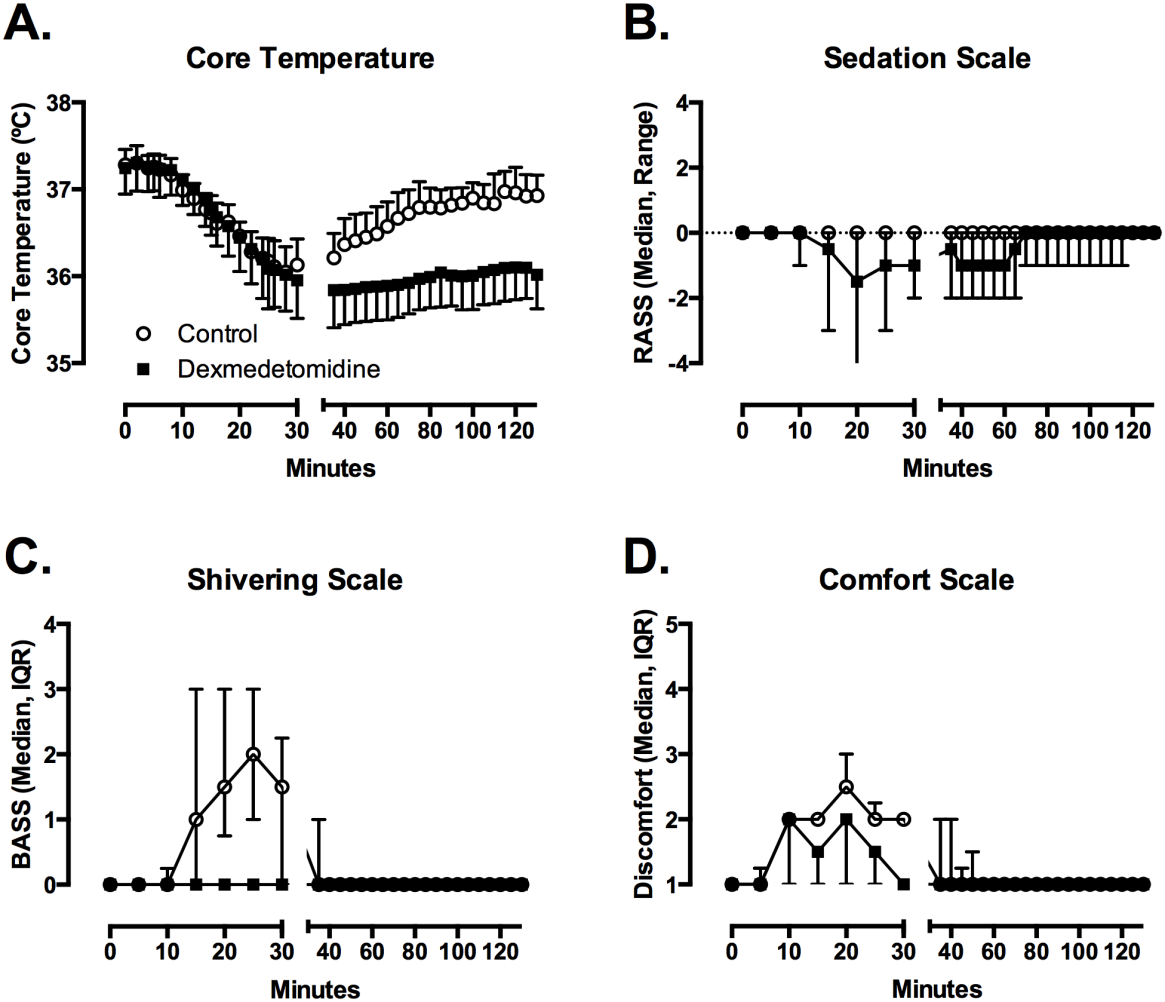
Dexmedetomidine suppresses the shiver threshold through moderate sedation, no shivering and improved comfort. Compared to saline infusion (circles), dexmedetomidine infusion allowed a more prolonged reduction in core temperature (A), with moderate sedation (B), no shivering (C), and slight improvement in comfort (D). Figures depict mean with SD of temperature, median with range for sedation, and median with interquartile range for shivering and comfort scales.

In all subjects, core body temperature decreased during cold saline infusion (Time, p = 0.005) (Fig 1). Temperatures were lower in the dexmedetomidine group (Drug, p<0.001), and drug altered the change in temperature over time (Drug x Time, p<0.001). The lowest temperature achieved for each subject ranged from 35.0°C to 36.3°C after dexmedetomidine (mean (SD) = 35.7 (0.4°C) and from 35.7°C to 36.5°C after placebo (mean (SD) = 36 (0.3°C) (t = 2.65; df = 9; p = 0.027). With placebo treatment, temperature began to increase immediately after the cold saline infusion was complete, and returned to >36.5°C by 85 minutes in all 10 subjects. With dexmedetomidine treatment, temperature remained below 36.3°C for 9 of the 10 subjects and below 36.0°C for 6 of the 10 subjects during the entire 120-minute observation period.

Shivering (BSAS>0) appeared at a mean (SD) core temperature of 36.6 (0.3°C (range 36.1–37.1°C) after placebo. Shivering was observed briefly in only 3 subjects after dexmedetomidine at a mean of 36.2 (0.4°C (range 35.9–36.7°C). Using a conservative assumption that the threshold for shivering in the 7 other subjects was the lowest temperature achieved during the study, we estimate that the mean shivering threshold after dexmedetomidine was 35.8 (0.4°C (range 35.0–36.7°C) (t = 4.28; df = 9; p = 0.002). We are confident that the actual shivering threshold was lower than this estimate, because no shivering was observed in 7 of 10 subjects.

**Fig 2:**
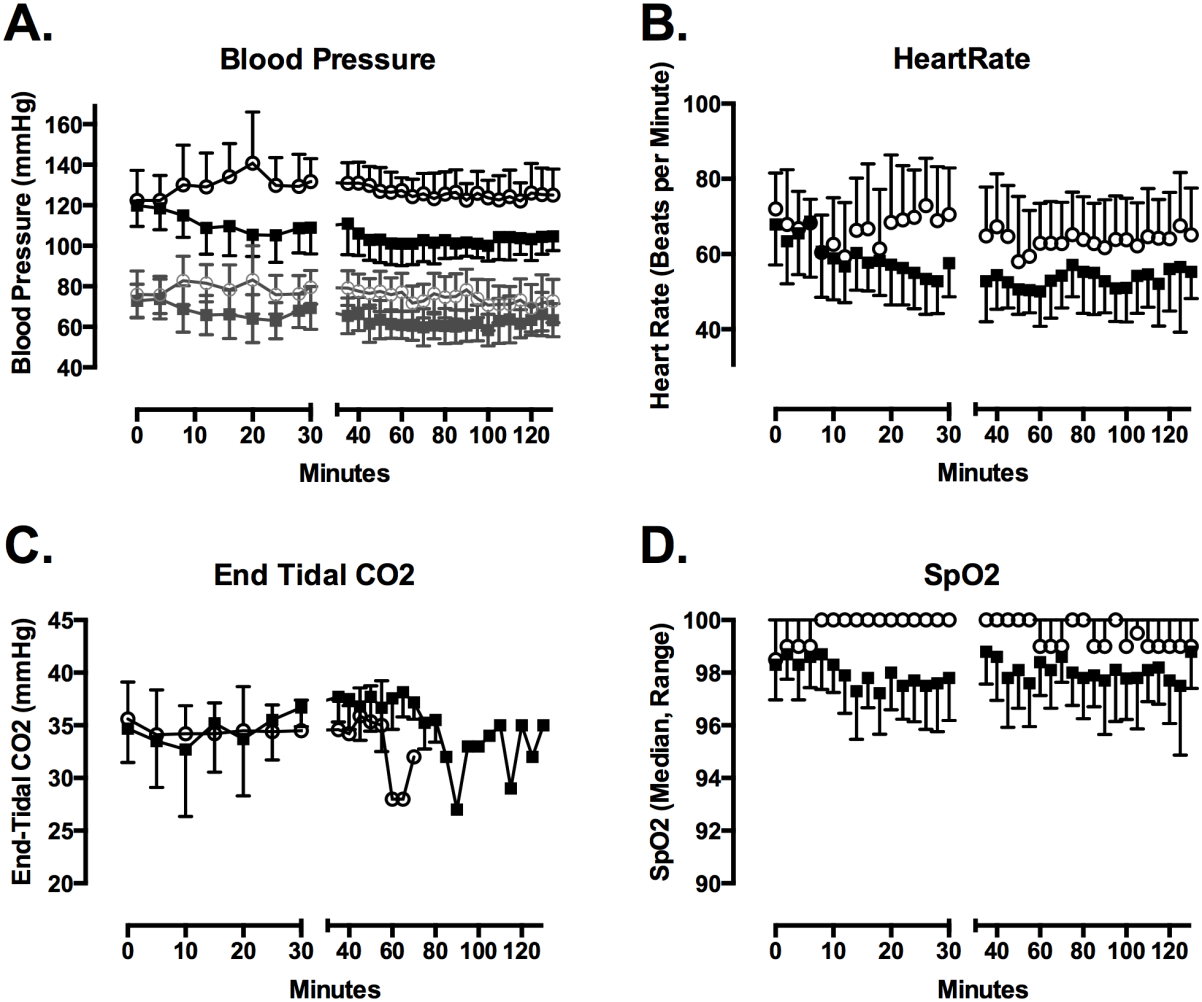
Dexmedetomidine infusion lowered SBP (A) and heart rate (B). In (A) systolic blood pressure is depicted in solid lines and symbols, while diastolic blood pressure is depicted in gray lines and symbols. End-tidal CO2 did not differ between groups (C). Pulse oximetry determined SpO2 was lower with dexmedetomidine, though the magnitude of the reduction was not clinically important (D). Figures depict mean with SD for blood pressure, heart rate and end tidal CO2. Median and range for SpO2 is depicted.

Heart rate (Drug, p <0.001), SBP (Drug, p<0.001), diastolic blood pressure (Drug, p<0.001), and respiratory rate (Drug, p<0.001) changed over time (Fig 2). The maximum reduction in SBP relative to baseline was greater (mean difference (95% confidence interval) = 26 (17–34) mmHg) after dexmedetomidine than after placebo (8 (2–15) mmHg) (t = 6.97; p = 0.0001). The maximum reduction in diastolic blood pressure (19 (9–27) mmHg and 14 (6–21) mmHg) and heart rate (23 (16–30) bpm and 25 (16–34) bpm) did not differ between dexmedetomidine and placebo days. Heart rate and blood pressure increased slightly during cold fluid administration in the placebo group. End-tidal CO2 did not differ between drug groups and did not vary over time. The lowest recorded SpO2 was lower after dexmedetomidine (median, IQR = 95.5%, 95–96%) than after placebo (97%, 96–97%) (p = 0.02).

Sedation (RASS<0) was evident in 9 of 10 subjects after dexmedetomidine (RASS from -1 to -3) and in 0 of 10 subjects after placebo. The total RASS (p = 0.0076) and lowest RASS (p = 0.0068) were lower after dexmedetomidine. Several subjects snored while sleeping, but no clinically significant respiratory compromise was noted. All subjects would awaken promptly to voice and could complete tasks. RASS scores returned to 0 in all 10 subjects by 90 minutes. However, most subjects reported feeling tired for several hours after drug infusion.

Total BSAS (p = 0.0049) and maximum BSAS (p = 0.0052) were lower after dexmedetomidine. Comfort and thermal sensation scales did not differ between groups. Subjects reported that the cold saline infusion into the arm was unpleasant, and described it as a cramping feeling in the muscles of the arm.

**Fig 3:**
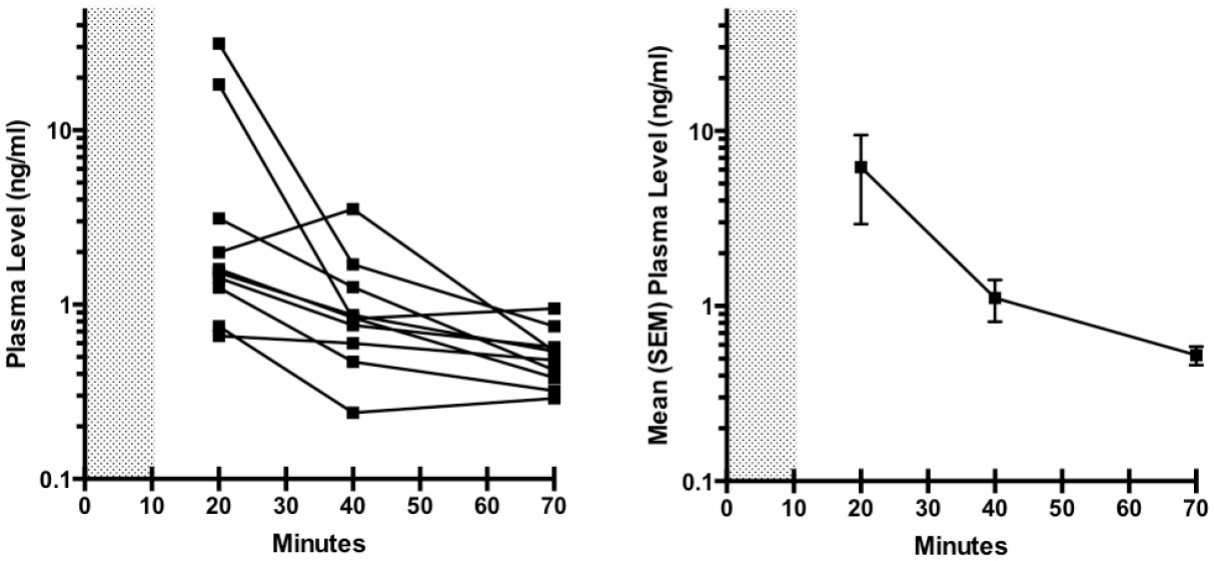
Dexmedetomidine plasma levels after bolus administration. Dexmedetomidine plasma levels in individual subjects (A), and mean with SEM levels for the entire group (B). After the 1 μg/kg bolus infusion, levels were 0.3 ng/ml or higher in all subjects for at least 60 minutes.

Individual and mean plasma dexmedetomidine levels over time are depicted in Fig 3. The two female subjects had notably higher dexmedetomidine concentrations at 10 minutes than the male subjects, but were not different at later time points. Plasma levels remained above 0.3ng/ml for all subjects for 60 minutes. When shivering was observed in three subjects receiving dexmedetomidine, the closest measured plasma concentrations and core temperatures were 1.26 ng/ml and 36.5°C, 0.95 ng/ml and 36.7°C, and 0.84 ng/ml and 36.1°C.

## Discussion

This study confirmed that a bolus of 1.0 μg/kg dexmedetomidine over 10 minutes reduces the shivering threshold by at least 0.8°C. This effect lasts at least 120 minutes. Drug infusion is associated with some reduction in heart rate and blood pressure. Sedation from this dose of dexmedetomidine is mild, lasts less than 90 minutes and produces no significant respiratory compromise. These data suggest that this drug and dosing may be useful clinically to as part of a protocol for rapid induction of protective hypothermia during acute stroke, myocardial infarction or other ischemic injury.

An interesting observation is that the cold saline infusion induced similar decrease in temperature for both placebo and dexmedetomidine days. These data suggest that the cold saline intervention is sufficiently strong and fast to overwhelm the initial shivering response, which takes some time to correct the core temperature. However, the rapid rebound of temperature on control visits replicates our observations and those of others that sustained reduction of temperature after cold fluid infusion requires pharmacological or physical maintenance measures [13, 14, 30, 31].

The suppression of shivering in this study is consistent with prior observations using continuous infusions of dexmedetomidine. In normal volunteers, shivering threshold decreased 2.4°C/ng/ml (to 34.7°C at 0.48ng/ml and to 34°C at 0.83 ng/ml) [16]. A separate study found shivering threshold decreased to 36°C in normal volunteers with plasma dexmedetomidine levels of 0.36 ng/ml [15]. We observed suppression of shivering threshold by at least 1.0°C with a mean 30-minute plasma dexmedetomidine level of 1.11ng/ml. This effect was achieved without respiratory depression. The present data together with prior studies suggest that target plasma levels of 1.0–1.5ng/ml might suffice to induce and maintain hypothermia of 1–2°C below the normal shivering threshold. One potential mechanism may be direct inhibition of central thermogenesis [32]

Dexmedetomidine plasma levels in this study are consistent with prior pharmacokinetic data in adult volunteers and in critically ill adult patients [33–37]. Estimates for the volume of distribution for dexmedetomidine are 199 L, 1.54 L/kg (107 L in a 70 kg subject), 173 L, 223 L and 108 L [33–37]. Dexmedetomidine is eliminated via hepatic metabolism, and estimates for clearance are 0.51L/min (30.6 L/hr), 8.9 ml/min/kg (37.4 L/hr in a 70 kg subject), 48L/hr, 39.7 L/hr, and 38 L/hr [33–37]. In critically ill adults the distributional t_1/2_ is about 8 minutes [37]. In previous studies, infusing 2.0 μg/kg dexmedetomidine over 5 minutes achieves peak plasma levels ~10 ng/ml immediately that decline to about 1 ng/ml within 10 minutes, and infusing 0.6 μg/kg over 10 minutes achieves plasma levels ~0.8 ng/ml that declined to ~0.3 ng/ml after the initial distribution [33–34]. Our infusion of 1.0 μg/kg over 10 minutes achieved peak levels ~6 ng/ml that declined to 1.1 ng/ml within 30 minutes, concentrations that are between the two prior studies. Limited data suggest that pharmacokinetic variability in healthy volunteers may result from genetic polymorphism in enzymes responsible for dexmedetomidine metabolism, such as CYP2A6, UGT1A4, and UGT2B10 [38]. The apparent slower t_1/2_ for distribution in our two female subjects is also an interesting incidental observation that would require further studies to confirm.

The primary side effects of dexmedetomidine were decreased SBP and sedation. The average 26 mmHg SBP decrease was not clinically important for normal volunteers. However, these changes might be significant for stroke patients with large vessel occlusions and blood-pressure dependent cerebral blood flow. Heart rate also declined an average 24 bpm approaching 40 bpm in several subjects, but this change did not differ between drug and placebo days. Our subjects were previously healthy, and so many had low resting heart rates at the beginning of the study, and the observed decline is probably related to temperature and fluid volume effects rather than drug. Another theoretical concern would be the effect of the fluid bolus on serum pH. Prior literature of rapid saline infusions to induce hypothermia have demonstrated a decrease in serum pH of 0.09 [39]. This small change should be normalized through respiratory compensation in healthy individuals, reducing any impact of this effect. After the drug bolus, all patients were sedated with a peak effect at about 20 minutes. Sedation did not result in airway compromise, and all subjects could be aroused with verbal prompting. This profile of side effects would be reasonable for patients with acute medical emergencies in whom temperature manipulation might be considered, because these critical patients would also be closely monitored during the same time period.

The synthetic opioid agonist meperidine is an alternative for treating shivering in awake patients, but has the opioid side effect of respiratory depression as well as potentially neurotoxic metabolites [15, 40]. Meperidine binds to multiple receptors, including alpha-2-receptors [41]. The suppression of shivering and thermogenesis of meperidine is not antagonized by naloxone in rats but is antagonized by the alpha-2-adrenoceptor antagonist atipamezole in mice [42, 43]. Furthermore, shivering threshold suppression by meperidine is additive with the suppression by dexmedetomidine, suggesting that these two drugs work via the same pathway [15]. In terms of efficacy, shivering threshold decreased to 36°C in normal volunteers with a meperidine plasma level of 0.33 ng/ml (~40 mg/hr), and decreased to 33.5°C at meperidine plasma concentrations of 1.8 ng/ml (~120 mg/hr) [15, 44]. However, the higher plasma level of meperidine also causes respiratory depression. Our results and others, suggest that dexmedetomidine may provide the same suppression of shivering afforded by meperidine without the opioid-receptor agonist side effects [15–17].

Future studies could determine the optimal dose of dexmedetomidine for maintenance of specific target temperatures. For example, a lower bolus dose than used in this study might allow similar reduction of temperature with less hypotension. In addition, continuous infusions of drug or repeated boluses may allow patients to maintain a reduced temperature for longer periods of time which may be desirable in some therapeutic settings. Clinically, dexmedetomidine is part of the clinical armamentarium used to prevent shivering in acute neurologic illness [45].

This study was limited by the failure of the cold saline infusion to reduce temperature further. Because seven subjects did not shiver at all after dexmedetomidine infusion, we could not determine the actual shivering threshold for these subjects. Six of ten subjects had decrease in temperature >1.0°C below the shivering threshold observed on placebo days. Our protocol did not allow additional fluid administration or additional cooling methods, and future studies should examine how deeply and for how long we can cool waking subjects. The small sample size of this trial is small and leaves us unable to determine a difference in gender response. Finally, the single-blind design is also a limitation.

## Conclusion

Bolus administration of 1.0 μg/kg dexmedetomidine decreases the average threshold temperature for shivering by at least 0.8° in normal volunteers. This effect lasts at least 90 minutes and is associated with a mean 26 mmHg reduction in SBP and moderate sedation, but no respiratory depression.
